# Clinical Profile and Pharmacological Management of Snakebites in Community Care Units: A Retrospective Study Using Two Military Hospital Databases in South Thailand

**DOI:** 10.3390/tropicalmed8070346

**Published:** 2023-06-29

**Authors:** Sethapong Lertsakulbunlue, Ratchakarn Suebtuam, Theethach Eamchotchawalit, Wittawat Chantkran, Janeyuth Chaisakul

**Affiliations:** 1 Department of Pharmacology, Phramongkutklao College of Medicine, Bangkok 10400, Thailand; sethapong.ler@pcm.ac.th; 2 Fort Thepsatrisrisunthon Hospital, Nakhon Si Thammarat 80310, Thailand; minkratchi@gmail.com; 3 Fort Wachirawut Hospital, Nakhon Si Thammarat 80000, Thailand; theethach.eam@pcm.ac.th; 4 Department of Pathology, Phramongkutklao College of Medicine, Bangkok 10400, Thailand; chantkran@gmail.com

**Keywords:** snakebites, Thailand, military hospitals, clinical profiles, treatment, antivenom

## Abstract

Snakebite envenoming is an occupational hazard in remote rural areas of South Thailand, where the highest incidence of snakebites is reported. In this work, a hospital-based retrospective study of snakebite patients from 2012 to 2022 at Fort Wachirawut Hospital and Fort Thepsatrisrisunthon Hospital, located in Nakhon Si Thammarat province, Thailand was conducted. Data from the laboratory investigation, physical examinations of snakebite victims, and clinical management, including pharmacological and non-pharmacological treatments, were evaluated. A total of 54 snakebite victims were included. The median age of patients was 49 years (IQR, 28 to 63). Males accounted for 74.1% of all participants. The majority of patients were bitten by Malayan pit vipers (68.5%), followed by unidentified snakes (18.5%), other non-venomous snakes (7.4%), and cobras (5.6%). The most common clinical manifestations were swelling (90.2%) and local pain (73.2%). One patient experienced respiratory failure following an envenoming by an unidentified venomous snake. No deaths were observed in this study. In total, 24 patients received antivenom administration (44.4%), most of whom were from Fort Wachirawut Hospital. Patients who were administered antivenom showed a median admission duration of three days (IQR, 3 to 4), compared with two days (IQR, one to three) for those who did not receive antivenom treatment (*p* < 0.001). In addition, paracetamol and prophylactic antibiotics, namely, amoxicillin-clavulanate and dicloxacillin, were the most common pharmacotherapies following snakebites. Overall, it was observed that these two community hospitals undertook appropriate clinical management under the standard guidelines for snakebite patients. This might be due to the effective emergency management, facilities, and clinical consultations. Finally, the management process in the medical teams also plays a crucial role in minimizing the severity of snakebite outcomes.

## 1. Introduction

Snakebite envenoming is a significant occupational hazard that primarily affects rural populations, resulting in a staggering number of deaths worldwide, particularly in Sub-Saharan Africa, South America, South Asia, and Southeast Asia [1]. In 2017, the World Health Organization (WHO) recognized snakebite envenoming as the highest priority neglected tropical disease [2]. The disease is responsible for a substantial burden of morbidity and mortality, prompting global efforts to reduce its incidence by 50% by 2030 [2,3]. A recent report highlighted the alarming impact of snakebite envenoming in the Association Southeast Asian Nations (ASEANs), estimating an annual death toll of 15,909 people, with 954 individuals requiring amputation as a result [4]. Notably, Malaysia exhibited the lowest incidence of snakebites, while Thailand boasted the lowest estimated mortality rate [4].

Despite snakebite envenoming not being the most pressing public health concern in Thailand, the southern region of the country reported the highest incidence of snake envenomation cases [5]. Importantly, this area is predominantly characterized by agricultural activities, with a significant portion of its land covered by rubber and oil palm plantations. The forested plantations in this region serve as favorable habitats for numerous venomous snakes found in the Malay Peninsula, including the Malayan pit viper (*Calloselasma rhodostoma*: MPV), the Malayan krait (*Bungarus candidus*), the King cobra (*Ophiophagus hannah*), and various cobra species, which include *Naja kaouthia* and *Naja sumatrana* [6]. These venomous snakes pose significant health risks, leading to adverse outcomes such as respiratory failure and skeletal muscle weakness due to the pre- and/or post-synaptic neurotoxins in cobra and krait venoms or excessive bleeding from pro-coagulant toxins in viper venoms.

Currently, the administration of specific snake IgG antibodies or snake antivenom is an acceptable treatment in alleviating snakebite envenoming outcomes. In cases of cobra, king cobra, or krait envenomation, the presence of skeletal muscle flaccid paralysis with respiratory failure serves as a clinical indicator for snake antivenom administration. On the other hand, antivenom is administered whenever abnormal bleeding associated with snake-venom-induced consumption coagulopathy (VICC) is observed following hematotoxic snake envenoming [6]. However, the administration of snake antivenom can become ineffective or cause hypersensitivity in some patients. This might be due to the use of inappropriate dosages, errors in snake identification, or the contamination of the antivenom during administration.

Nakhon Si Thammarat, located in the southern part of Thailand, has emerged as a prominent area for snake envenomation [7]. There are two community hospitals operated under the Royal Thai Army (RTA). According to a categorization by the ministry of public health (Thailand) based on the total number of hospital beds [8], Fort Wachirawut is categorized as a service-level “S hospital” (150 beds), while Fort Thepsatrisrisunthon Hospital is classified as a service-level “F2 hospital” (60 beds). These two community hospitals are located in a district with a high incidence of snakebite cases. Hence, they undoubtedly have a responsibility to offer treatment for snakebite victims with effective emergency management in order to minimize the incidence of morbidity and mortality. We, therefore, determined the clinical profiles and management of snakebite patients by analyzing data obtained from these two community hospitals. This study demonstrates post-hospital management strategies encompassing physical and laboratory examinations, pharmacotherapy, snake antivenom administration, and the availability of snake antivenom. Our findings will enhance understanding and knowledge of snakebite management by shedding light on the capacity of community care units in handling snakebite cases and emergency pre-/post-hospital treatment.

## 2. Materials and Methods

### 2.1. Study Design and Subjects

The study was conducted using data from patient record forms during the period from 1 January 2012 to 30 June 2022. We employed a cross-sectional study design to ascertain the prevalence and management of patients afflicted with snakebites who sought medical attention at Fort Wachirawut hospital and Fort Thepsatrisrisunthon Hospital, Nakhon Si Thammarat. Both hospitals provide healthcare services not only to military personnel and their family but also to other Thai civilians [8,9]. The data of patients with a snakebite diagnosis who were admitted to either hospital were eligible for analysis.

### 2.2. Data Collection

After obtaining permission from the directors of the enrolled hospitals, data were retrieved from the medical records using a case record form consisting of three categories: (1) patient characteristics and demographic data such as age, gender, and visit date, along with the time of arrival at the emergency department (ED), the time of the snakebite, the bite site, the snake type, and pre-hospital management. (2) Laboratory tests performed at the ED such as the complete blood count (CBC), serum electrolytes, serum creatinine (Cr), the whole-blood clotting time (WBCT), the venous clotting time (VCT), and the international normalized ratio (INR); follow-up WBCT and VCT values were also recorded. (3) Information regarding management, envenoming outcomes, clinical manifestations, admission time, antivenom treatment, and pharmacological treatments while staying at the hospital. Snakebites were identified using the International Classification of Diseases, Tenth Revision (ICD-10) codes T63.0, as documented in the medical records. Additionally, the appropriateness of antivenom administration was reviewed and retrieved from the medical records.

To evaluate the effectiveness of snake antivenom administration to snakebite patients, we determined the amount of antivenom utilized in each hospital and the criteria for determining eligible patients who required antivenom administration. These criteria were based on the indications recommended by the Queen Saovabha Memorial Institute (QSMI), which is the antivenom manufacturer and also provides a list of its recommended uses. Indications for antivenom treatment in hematotoxic snake envenoming comprises (1) a VCT greater than 20 min; (2) an unclotted 20 min WBCT (20WBCT); (3) a platelet count less than 50 × 10^9^/L; (4) an INR greater than 1.2; (5) systemic bleeding; (6) compartment syndrome requiring fasciotomy. The recommended dose for hematotoxic snake envenoming is 3–5 vials per dose, and the duration of infusion is 30–60 min [6,10]. For neurotoxic snake envenomation, the indications of antivenom administration include signs of muscle weakness and known envenoming from the banded krait (*Bungarus fasciatus*) or Malayan krait (*B. candidus*). The recommended dose is 5–10 vials per dose, and the duration of infusion is 30–60 min [10].

An elevated creatinine phosphokinase (CPK) level is defined as 200 U/L or above [11]. The serum sodium range is typically considered normal within the range of 135–145 mmol/L, while potassium is within the normal range of 3.6–5.1 mmol/L, chloride is within the range of 96–106 mmol/L, and bicarbonate is normally within 22–29 mmol/L [12].

### 2.3. Statistical Analysis

Data analysis was conducted using the Statistical Package for the Social Sciences (SPSS, Inc., Chicago, IL, USA) for Windows, version 28.0. The normality of the data was assessed using the Kolmogorov–Smirnov test. Descriptive statistics were employed to analyze baseline characteristics, with continuous data presented as the mean and standard deviation (SD) or median and interquartile range (IQR) and categorical data presented as frequency and percentage. The Student’s *t*-test was utilized to compare continuous variables, while the Mann–Whitney U-test was used when appropriate. Categorical variables were compared using Pearson’s chi-squared test as indicated. Statistical significance was determined using a two-sided *p*-value of less than 0.05.

## 3. Results

### 3.1. Baseline Characteristics of Participants

**Figure 1:**
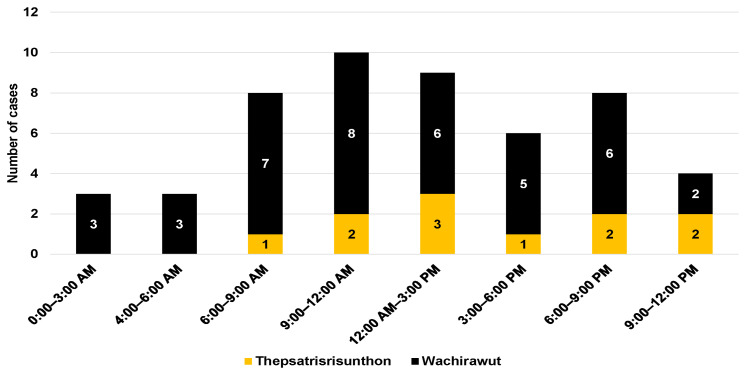
Snakebites by time of day.

A total of 54 patients participated in this study including 41 patients from Fort Wachirawut hospital and 13 patients from Fort Thepsatrisrisunthon hospital. Most patients (96.3%) visited the ED directly, while only two (3.7%) were referred to the ED from another hospital. The median age of the participants was 49 years (IQR, 28 to 63) (Table 1). Of all participants, 40 patients (74.1%) were male. More than two-thirds of the participants were bitten during the day by MPV (Figure 1). Almost all patients presented to the ED with a fang mark, with 53.7% and 25.9% having fang marks on their feet and hands, respectively. Only one case was bitten around the pectoral region (chest). Over 90% of the bite marks were swollen, and 73.2% of the participants reported experiencing local pain. Moreover, six patients (14.6%) reported local hematomas and one participant was diagnosed with compartment syndrome during their ED visit.

**Figure 2:**
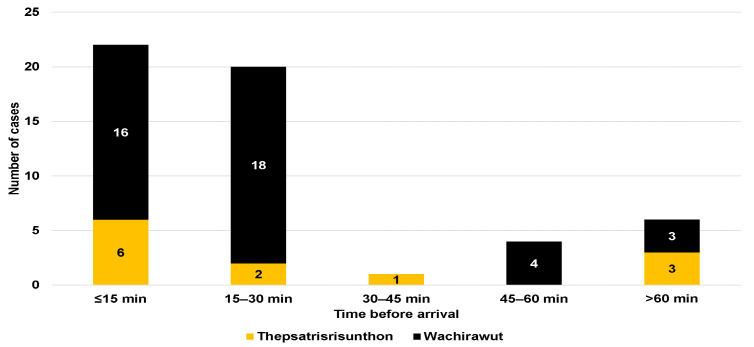
Time before arriving at the emergency department of Fort Thepsatrisrisunthon Hospital and Fort Wachirawut Hospital for snakebites.

**Figure 3:**
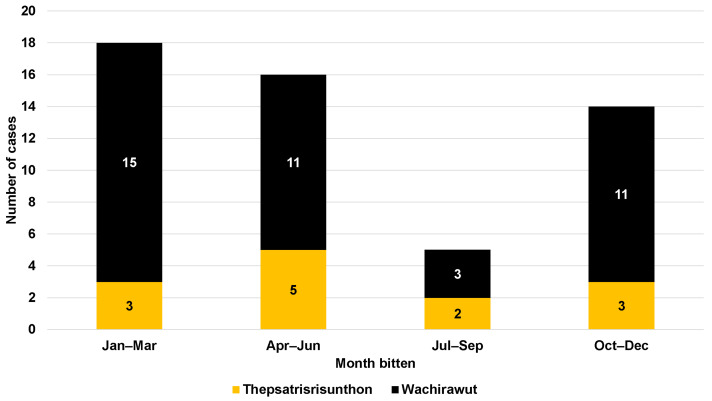
Seasonal cases of snakebite patients admitted to Fort Thepsatrisrisunthon Hospital and Fort Wachirawut Hospital.

Regarding neurological symptoms, only one patient from Fort Wachirawut hospital reported muscle weakness, ptosis, and respiratory failure (Table 1). The majority of snakebite victims arrived at the hospital within an hour of being bitten (88.7%), with a median time of 20 min (IQR, 15 to 30) for Fort Wachirawut hospital and 17.5 min (IQR, 10 to 140) for Fort Thepsatrisrisunthon hospital (Figure 2). Notably, 22 patients arrived within 15 min of being bitten, and 20 patients arrived within 15–30 min. Figure 3 shows that around two-thirds of the study participants were bitten in the period from January to June, with only 9.3% being bitten from July to September.

Regarding the laboratory results obtained during the patients’ first visit to the ED, hyponatremia was observed in four individuals (7.4%), three of whom were bitten by MPVs, and one by an unknown snake. Additionally, hypokalemia was detected in nine patients (16.7%), with seven of them bitten by MPVs and two by unknown snakes. Among the five patients who underwent serum CPK (creatine phosphokinase) testing, one patient bitten by a MPV showed an elevated CPK level of 310 U/L, while another patient, who was bitten by an unknown snake, had a serum CPK level of 217 U/L.

### 3.2. Characteristics of Patients Stratified by Antivenom Treatment and Clinical Management

In total, 24 patients received snake antivenom (44.4%) (Table 2). Patients who received antivenom treatments arrived at the ED at a median time of 17.5 min (IQR, 10 to 30) after being bitten; in comparison, those who did not receive antivenom treatment had a median time of 20 min (IQR, 15 to 40). The duration of admission was longer for patients who received antivenom treatment, with a median time of three days (IQR, 3 to 4), compared with those who did not receive antivenom treatment, with a median of two days (IQR, 1 to 3) (*p* < 0.001). Additionally, mean platelet levels were lower in the antivenom group (211 × 10^9^/L) than in the non-antivenom therapy group (257 × 10^9^/L), although this difference did not reach statistical significance (*p* = 0.053). Hyponatremia was observed at similar levels in both the antivenom (9.5%) and non-antivenom treatment groups, while a higher proportion of hypokalemia was observed among the antivenom group (28.6%) compared to the non-antivenom treatment group (13.0%).

Of these 24 antivenom treatment cases, 23 patients with hematotoxic activity were treated by the administration of MPV monovalent antivenom, with a median of five vials per patient (IQR, 3.0–7.0; 22 cases) and Hemato Polyvalent antivenom (three vials in one case). Meanwhile, 20 vials of Neuro Polyvalent AV were administered to a patient envenomed by an unidentified venomous snake with neurotoxic outcomes. This patient was intubated and admitted for 19 days. The primary indications for antivenom administration were applied in 9 patients showing prolonged VCT and 11 patients with unclotted 20 min WBCTs (Table 3). Overall, 22 patients (91.3%) received the antivenom with the appropriate indication and dosage, while 2 envenomed patients were given antivenom without an indication (Table 3). Adverse reactions including itchiness and skin rashes were reported in five patients (20.8%) following the administration of MPV monovalent antivenom (3–10 vials).

### 3.3. Pharmacological Treatments and Other Treatments of Patients with Snakebites

Paracetamol (83.3%) and other NSAIDs (7.4%) including ibuprofen and diclofenac were prescribed. In addition, pethidine was prescribed for a patient with severe pain and impending compartment syndrome. Antibiotics were prescribed to 49 patients (90.7%), and all patients from Fort Wachirawut Hospital received amoxicillin–clavulanic acid (Table 4). Tetanus toxoids were administered to 41 patients, while corticosteroids were not used. Notably, information regarding pre-hospital management, i.e., the tying of a tourniquet, mouth suction, and traditional healers, was not reported.

## 4. Discussion

Snakebite envenoming is a neglected tropical disease that leads to various severe clinical outcomes. Swift prehospital management, encompassing first-aid measures and resuscitation, and the thorough physical and laboratory assessments, including the administration of antivenom or other pharmacological treatments, is imperative to prevent fatalities and mitigate disabilities. In fact, timely prehospital management, i.e., snakebite first aid, appears to be an important tool to minimize the severity of clinical manifestations by reducing the systemic absorption of snake venom.

In the present study, a total of 54 snakebite patients were admitted to two community hospitals located in Nakhon Si Thammarat during the period from January 2012 to June 2022. It is important to acknowledge that the sample size in this study is relatively small, considering the existence of larger public health general hospitals in the province. Additionally, it should be noted that, although the service capacity of Fort Wachirawut Hospital falls under the category S service level [8,9], it has the potential to accommodate only up to approximately 150 inpatients. In fact, the actual inpatient capacity at Fort Wachirawut Hospital is approximately 90 beds. In addition, Fort Wachirawut Hospital exhibits a greater inpatient capacity than Fort Thepsatrisrisunthon Hospital, which operates at an equivalent category F2 service level but has only 30 beds in the inpatient department [9]. Nonetheless, both hospitals are equipped to provide antivenom therapy and effectively manage cases of venomous snakebites.

The incidence of snakebites was observed to occur throughout the year, with the lowest number of cases reported during the period from July to September. However, a previous study showed that the peak of the snakebite season was in May, which corresponds to the early monsoon season [5,7]. This finding may be attributed to the geographic location of Nakhon Si Thammarat, which is situated in the Malay peninsula and is influenced by both the south-western and north-eastern monsoons, resulting in a rainy climate throughout the year. Indeed, the high incidence of snakebite cases are related to agricultural practices, e.g., soil preparation, planting, and harvesting, especially in the rainy season. Moreover, we also observed the highest prevalence of snakebites during the period of 2019–2022. This increase can be attributed to the COVID-19 pandemic, during which Thai people were restricted from visiting urban areas and prolonged their stay in rural areas.

Most of the participants were bitten during the morning hours, between 6:00 a.m. and 12:00 p.m., which are the working hours for tapping rubber trees [13]. Almost every patient arrived at the emergency department within 60 min of sustaining a snakebite, with a median time of 20 min. In contrast, a previous study reported longer median times before arrival of 40 and 60 min [14]. The mean time from the bite to emergency department was even longer (175 min) in the study conducted by Wongtongkam et al. (2005) [7]. This may be because those snakebite data were collected in remote rural areas.

In this study, we encountered only one patient who presented neurotoxic activity following envenomation by an unidentified snake. This patient received two separate doses of the Neuro Polyvalent antivenom, with each dose consisting of 10 vials. The administration of antivenom was necessitated by the worsening neurotoxic symptoms [6], then the patient was subsequently intubated at Fort Wachirawut Hospital. In fact, the polyvalent antivenom is more challenging and expensive to produce. As a result, it should be reserved for cases when the species of responsible snakes is unknown. This Neuro Polyvalent antivenom contains neutralizing effect against major venomous neurotoxic snakes in Thailand. These include cobras (*N. kaouthia*), king cobras (*O. hannah*), banded kraits (*B. fasciatus*), and Malayan kraits (*B. candidus*) [6]. The neurotoxicity following envenoming by these species can be skeletal muscle weakness, ptosis, dysphagia, and respiratory failure. These outcomes are induced by the rapidly absorbed neurotoxins that inhibit the binding activity between nicotinic acetylcholine receptors and the neurotransmitter “acetylcholine (ACh)” at the neuromuscular junction [6]. Moreover, it is very important to delay systemic absorption of the venom by using elastic bandages to immobilize the site of the bite especially the lower and upper limbs (the bitten limb). As these bitten sites potentially accelerate the systemic diffusion of snake venom [6]. In addition to these neurotoxic effects, cobra venom also induces potent cytotoxic effects, resulting in tissue necrosis and compartment syndrome [6,15].

In line with previous studies conducted in the southern region of Thailand, MPV has become a common venomous snake, the most prevalent envenoming snake in the Malay peninsula [5,7,10,13]. The clinical manifestations following MPV envenoming ranged from local (e.g., swelling, blistering, and bleeding at the bite sites) to systemic effects, especially venom-induced consumption coagulopathy leading to hemorrhagic syndromes [6,7]. Our findings showed that 62.2% of patients who were bitten by MPVs exhibited coagulopathies. The proportion of patients with coagulopathies in this study was greater than those in previous studies conducted by Wongtongkam et al. (2005; 46.4%) [7], Kraisawat et al. (2020; 38.6%) [13], and Viravan et al. (1992; 33.8%) [14]. This phenomenon could be attributed to the fact that different laboratory parameters were recorded in the present study, and a variety of laboratory cutoffs were used; for example, Wongtongkam et al. (2005) used a period of 30 min as a threshold for prolonged VCT [7], whereas a period of 20 min was used to determine abnormal blood coagulation in our study.

Moreover, a slight increase in CPK was detected in two cases of MPV envenoming. This would be a concern, with CPK levels greater than 1000 U/L suggesting a risk of acute kidney injury (AKI). Indeed, many Asian viper venoms comprise toxic compositions associated with nephrotoxicity, i.e., snake venom metalloproteinases (SVMP) and phospholipase A_2_ (PLA_2_). These two enzymes were demonstrated to induce pathophysiological changes in the kidney following the experimental envenoming with viper venoms [16,17]. In this study, CPK evaluation was conducted in only five cases of snake envenoming. It should be kept in mind that AKI can be induced following Asian viper envenomation [18]. Hence, CPK level and other routine laboratory evaluations for renal function, e.g., blood urea nitrogen (BUN), creatinine and creatinine clearance, including renal imaging should be performed, if these were applicable in patients who have a high risk of rhabdomyolysis, such as patients envenomed by Russell’s vipers (*Daboia* spp.), Malayan pit viper (*C. rhodostoma*), and sea snakes (*Hydrophis* spp.).

Almost all systemic effects observed following snake envenoming can be effectively relieved by the administration of species-specific antivenoms. Indeed, snake antivenoms are produced by repetitive injection of venom into animals (usually horses or sheep). Therefore, they are polyclonal antibody mixtures with an affinity for the different antigenic components in the venom. Basically, snake antivenoms are classified as (1) monovalent antivenoms, which are prepared by the injection of venom from only one species of snake or (2) Polyvalent antivenoms which are produced by the injection of different snake venoms. Monovalent antivenoms have a low volume of specific antibodies for the snake species involved. Polyvalent antivenoms are more cost-effective to manufacture and are a better option for snakebite patients in order to minimize the problem of incorrect antivenom application due to diagnostic error. In Thailand, The Queen Saovabha Memorial Institute of the Thai Red Cross Society is the only snake antivenom manufacturer to produce mono-specific snake antivenoms, Neuro Polyvalent, and Hemato Polyvalent antivenoms for treating envenoming from the most significant venomous snakes in Southeast Asia. The QSMI also provides a comprehensive list of indications and dosages for the use of antivenoms [5,6]. However, inappropriate administrations of antivenom are still reported in Thailand. This can result in hypersensitivity reactions and is detrimental to both the patient and the hospital [5,6]. Tangtrongchitr et al. (2021) [5] demonstrated that 85.4% of patients with MPV envenomation who reported to the poison center received appropriate antivenom administration in terms of the dose and criteria [5]. Similarly, our study revealed that 91.7% (22 of 24 patients) received appropriate antivenom administration, as recommended by the QSMI.

In the present study, there were two patients who met the indication for antivenom treatment but did not receive it. The first patient was bitten by a MPV and had a prolonged INR of 1.29. This may be attributed to the marginal increase in the INR compared to another case that received antivenom treatment, which had a higher INR (1.65). The elevated INR was the sole indication for antivenom therapy in that particular case. The second patient was bitten by an unknown snake and had a prolonged INR (1.26) and a prolonged VCT (26 min and 27 min for the first and second follow-up examinations, respectively). Despite the availability of MPV monovalent antivenom, the on-duty staff decided to monitor the patient’s clinical manifestations and VCT for a day until they returned to normal, after which the patient was discharged. Although the increase of INR is marginal, it is important to note that an INR value above 1.2 has a sensitivity of 85.7% and a specificity of 95.6% for diagnosing a fibrinogen level below 100 mg/dL, which is an alternative test for evaluating coagulopathy [19]. In contrast, there were two cases where antivenom was administered without a specific indication due to the presence of extensive local hematoma without the diagnosis of compartment syndrome. However, document indicating improvement following antivenom administration in these cases was not available.

Overall, the administration of antivenom in these two fort hospitals appears to adhere to standard indications. This could be attributed to the consultation process that involves discussions between internists and internal medicine specialists for managing non-emergency procedures, i.e., antivenom administration, intubation, and fasciotomy. This collaborative approach is likely to contribute to more accurate diagnoses and treatment decisions. Moreover, it is noteworthy that both the on-duty specialists and interns at our hospital graduated from the same military medicine college, fostering a favorable consultation environment. Collaborative consultation approaches have been demonstrated to reduce misdiagnosis, improve patient outcomes, and enhance patient satisfaction and safety [20]. Additionally, since fewer patients seek care at fort hospitals compared to public health hospitals, patients at fort hospitals receive more individualized attention, which may also contribute to better outcomes. Furthermore, the presence of readily available emergency department specialists also improves the effective patient management.

In the current study, prophylactic antibiotics such as amoxicillin–clavulanic acid and dicloxacillin were administered to most patients. However, the routine use of antibiotics in snakebite envenoming is controversial. In the Thai clinical practice guidelines, amoxicillin and clavulanic acid are recommended only in the presence of infection [10]. However, previous reports showed the benefits of amoxicillin–clavulanate, especially in the prevention of secondary infections following snakebites, due to the resistance of bacterial species commonly found in snakebite sites and snake oral microbiota to β-lactam antibiotics [21,22]. Indeed, third-generation cephalosporins were also reported to be generally effective in snakebite cases [22]. Therefore, prophylactic antibiotic administration should only be considered in patients with severe local signs of envenomation, while empirical antibiotic administration should be considered in patients with local or systemic signs of infection, irrespective of the degree of envenomation. The most effective empirical antibiotics are third-generation cephalosporins, while amoxicillin–clavulanate may no longer be the best option [23]. Nevertheless, further research is needed to identify the bacterial species responsible for infections in snakebite wounds in Thailand.

Regarding other treatments, both fort hospitals provided standard patient management. Corticosteroids were not administered to any of the patients as none of the patients experienced severe allergic reactions following antivenom administration. Moreover, previous clinical trials also reported that treatment with adjunctive corticosteroids did not contribute to positive clinical outcomes in snakebite cases [10]. As snakebites potentially cause a tetanus-prone wound. Therefore, the tetanus toxoid was administered to some snakebite patients who were not vaccinated in the past five years. We also found that delayed tetanus toxoid vaccinations were applied in some patients with coagulopathy. As the intramuscular administration of tetanus toxoids may increase the risk of excessive bleeding [10,24]. In terms of pre-hospital management, none of the patients reported tying tourniquets, mouth-sucking the wound, or employing traditional remedies prior to seeking medical care, which potentially caused serious complications such as compartment syndrome or accelerating venom distribution.

Finally, some of this study’s limitations should be noted. As a cross-sectional study, data facilitating further evaluations of the long-term outcomes after treatment might be difficult to obtain. In addition, there may be some missing details, such as the time before arrival at the emergency department, clinical manifestations, and diagnosis, which depend on the individual decisions of primary physicians at the emergency department. Missing patient information and lost data in some periods of the study were found which might be due to the different hospital recording systems. Therefore, caution should be exercised in interpreting the findings and generalizing the information.

## 5. Conclusions

This study examines the clinical profiles and management of snakebites in two community hospitals. We found that the accessibility of effective treatment for snakebite patients was positively influenced by the staff’s knowledge of either pre- or post-hospital management, the doctor’s diagnosis, and the health scheme in each hospital. The management of snakebites should be standardized among all staff and physicians. To decrease the inappropriate antivenom treatment, a detailed standing order or protocol could be available at the emergency department. Furthermore, there is a need for a revision of the antibiotic protocol for snakebites in these hospitals. It is also recommended to conduct pre-clinical studies to identify the bacterial species responsible for infections in snakebite wounds. Our findings suggest that updating clinical training for physicians dealing with snakebite patients will contribute to the appropriate and accurate administration of antivenom and other relevant treatments.

## Figures and Tables

**Table 1:** Characteristics of snakebite patients in two fort hospitals of Nakhon Si Thammarat (*n* = 54).

Characteristics	Thepsatrisrisunthon	Wachirawut	Total
	*n* (%)	*n* (%)	*n* (%)
Year			
2012–2015	2 (3.7)	3 (5.6)	5 (9.3)
2016–2019	3 (5.6)	14 (25.9)	17 (31.5)
2020–2022	8 (14.8)	24 (44.4)	32 (59.3)
Age (years)			
10–29	9 (16.7)	6 (11.1)	15 (27.8)
30–49	3 (5.6)	11 (20.4)	14 (25.9)
50–69	1 (1.9)	19 (35.2)	20 (37)
>70	0 (0)	5 (9.3)	5 (9.3)
Median (IQR)	25 (23–30)	57 (42–65)	49 (28–63)
Gender			
Male	9 (16.7)	31 (57.4)	40 (74.1)
Female	4 (7.4)	10 (18.5)	14 (25.9)
Admitted/Referred			
Emergency department	13 (24.1)	39 (72.2)	52 (96.3)
Referred	0 (0)	2 (3.7)	2 (3.7)
Fang mark	13 (24.1)	40 (74.1)	53 (98.1)
Bitten area			
Arm	0 (0)	2 (3.7)	2 (3.7)
Hand	2 (3.7)	12 (22.2)	14 (25.9)
Leg	0 (0)	8 (14.8)	8 (14.8)
Foot	10 (18.5)	19 (35.2)	29 (53.7)
Chest	1 (1.9)	0 (0)	1 (1.9)
Admission duration (day)			
Median (IQR)	1 (0–2)	3 (3–4)	3 (2–4)
Snake type			
Malayan pit viper (*Caloselasma rhodostoma)*	3 (5.6)	34 (63.0)	37 (68.5)
Cobra (*Naja* spp.)	2 (3.7)	1 (1.9)	3 (5.6)
Other non-venomous snakes	4 (7.4)	0 (0)	4 (7.4)
Unidentified species	4 (7.4)	6 (11.1)	10 (18.5)
Clinical Manifestation * (*n* = 41)			
Local pain	6 (66.7)	24 (75.0)	30 (73.2)
Swelling	6 (66.7)	31 (96.9)	37 (90.2)
Local hematoma	4 (44.4)	2 (6.3)	6 (14.6)
Bleb	0 (0)	2 (6.3)	2 (4.9)
Compartment syndrome	0 (0)	1 (3.1)	1 (2.4)
Muscle weakness	0 (0)	1 (3.1)	1 (2.4)
Respiratory failure	0 (0)	1 (3.1)	1 (2.4)
Ptosis	0 (0)	1 (3.1)	1 (2.4)

* Some documented data were missing and only 41 patients had their clinical manifestation documented at the emergency department.

**Table 2:** The association between patient characteristics/laboratory results and snake antivenom administration in envenomed patients at Fort Thepsatrisrisunthon Hospital and Fort Wachirawut Hospital.

Characteristics	Did Not Receive Antivenom	Received Antivenom	*p*-Value
	*n* (% of 30 Patients)	*n* (% of 24 Patients)	
Year			0.027 ^a^
2012–2015	4 (13.3)	1 (4.2)	
2016–2019	5 (16.7)	12 (50.0)	
2020–2022	21 (70.0)	11 (45.8)	
Hospital			0.002 ^a^
Wachirawut	18 (60.0)	23 (95.8)	
Thepsatrisrisunthon	12 (40.0)	1 (4.2)	
Age (years)			
Median (IQR)	45.5 (27.0–63.0)	51.5 (33.5–63.0)	0.433 ^b^
Sex			0.445 ^a^
Male	21 (70.0)	19 (79.2)	
Female	9 (30.0)	5 (20.8)	
Time before arrival (min)			
Median (IQR)	20 (15–40)	17.5 (10-–0)	0.385 ^b^
Admission duration			
Median (Min–max)	2 (0–5)	3 (1–19)	<0.001 ^b^
INR (*n* = 36)			
Median (IQR)	1 (1.0–1.1)	1 (0.9–1.1)	0.325 ^b^
Platelet (×10^9^/L) (*n* = 50)			
Mean ± SD	257 ± 71.5	211 ± 91.7	0.053 ^c^
Serum creatinine (*n* = 49)			
Mean ± SD	0.9 ± 0.2	0.9 ± 0.2	0.553 ^c^
Serum sodium (mmol/L) (*n* = 44)			0.924 ^a^
Hyponatremia (<135)	2 (8.7)	2 (9.5)	
Normal (135–145)	21 (91.3)	19 (90.5)	
Hypernatremia (>145)	N/A	N/A	
Serum potassium (mmol/L) (*n* = 44)			0.202 ^a^
Hypokalemia (<3.5)	3 (13.0)	6 (28.6)	
Normal (3.5–5.1)	20 (87.0)	15 (71.4)	
Hyperkalemia (>5.1)	N/A	N/A	
Serum chloride (mmol/L) (*n* = 44)			0.905 ^a^
Hypochloremia (<96)	N/A	N/A	
Normochloremia (96–106)	20 (87.0)	18 (85.7)	
Hyperchloremia (>106)	3 (13.0)	3 (14.3)	
Serum bicarbonate (mmol/L) (*n* = 44)			0.260 ^a^
Hypobicarbonatemia (<22)	4 (17.4)	2 (9.5)	
Normal (22–29)	19 (82.6)	17 (81.0)	
Hyperbicarbonatemia (>29)	N/A	2 (9.5)	
Serum CPK (U/L) (*n* = 5)			0.833 ^a^
Normal	1 (100.0)	2 (50.0)	
Elevated (>200)	0 (0.0)	2 (50.0)	
Clinical Manifestation (*n* = 41)			
Local pain	14 (63.6)	16 (84.2)	0.138 ^a^
Swelling	19 (86.4)	18 (94.7)	0.368 ^a^
Local hematoma	4 (18.2)	2 (10.5)	0.489 ^a^
Bleb	1 (4.5)	1 (5.3)	0.915 ^a^
Compartment syndrome	0 (0.0)	1 (5.3)	0.276 ^a^
Muscle weakness	0 (0.0)	1 (5.3)	0.276 ^a^
Respiratory failure	0 (0.0)	1 (5.3)	0.276 ^a^
Ptosis	0 (0.0)	1 (5.3)	0.276 ^a^

CPK: creatinine phosphokinase, INR: international ratio, ^a^: Chi-square test, ^b^: Mann–Whitney U test, ^c^: independent *t*-test.

**Table 3:** The indications for antivenom treatment, antivenom type, and adverse reaction following antivenom administration among patients who were admitted to Fort Thepsatrisrisunthon Hospital and Fort Wachirawut Hospital (*n* = 54).

Characteristics	Thepsatrisrisunthon	Wachirawut
	*n* (%)	*n* (%)
Number of administered patients	1 (1.9)	23 (42.6)
Indications		
Prolonged VCT	0 (0)	9 (16.7)
Unclotted 20 WBCT	1 (1.9)	10 (18.5)
Platelet <50 × 10^9^/L	0 (0)	0 (0)
INR >1.2	N/A	4 (7.4)
Systemic bleeding	0 (0)	2 (3.7)
Compartment syndrome	0 (0)	2 (3.7)
Antivenom type		
MPV monovalent AV	1 (1.9%: 3 vials)	21 (38.9%: 3–20 vials/person)
Hemato polyvalent AV	0 (0)	1 (1.9%: 3 vials)
Cobra monovalent AV	0 (0)	0 (0)
Neuro polyvalent snake AV	0 (0)	1 (1.9%: 20 vials)
Appropriate administration (*n* = 24)		
With indication	1 (100.0% of 1 patients)	21 (91.3% of 23 patients)
Given with no indication	N/A	2 (8.7% of 23 patients)
Appropriate dosage	1 (100.0% of 1 patients)	23 (100.0% of 23 patients)
Adverse reactions		
Itchiness	0 (0)	2 (8.7% of 23 patients)
Rash	0 (0)	3 (13.0% of 23 patients)

VCT: venous clotting time, WBCT: whole-blood clotting time, INR: international ratio, AV: antivenom, MPV: Malayan pit viper.

**Table 4:** Pharmacological treatment of patients with snakebites (*n* = 54).

Characteristics	Thepsatrisrisunthon	Wachirawut
	*n* (%)	*n* (%)
Pain control		
Paracetamol	8 (14.8)	37 (68.5)
Other NSAIDs	2 (3.7)	2 (3.7)
Pethidine	0 (0.0)	1 (1.9)
Other (Orphenadrine + paracetamol)	3 (5.6)	0 (0.0)
Antibiotics		
Amoxicillin/Clavulanic acid	3 (5.5)	41 (75.9)
Dicloxacillin	5 (9.3)	0 (0.0)
Tetanus toxoid (dT/TT)	3 (5.5)	38 (70.4)

## Data Availability

The datasets used and/or analyzed in this study are available from the author on reasonable request.
